# Mathematical Modelling and Tuberculosis: Advances in Diagnostics and Novel Therapies

**DOI:** 10.1155/2015/907267

**Published:** 2015-03-15

**Authors:** Alice Zwerling, Sourya Shrestha, David W. Dowdy

**Affiliations:** Johns Hopkins Bloomberg School of Public Health, Baltimore, MD 21205, USA

## Abstract

As novel diagnostics, therapies, and algorithms are developed to improve case finding, diagnosis, and clinical management of patients with TB, policymakers must make difficult decisions and choose among multiple new technologies while operating under heavy resource constrained settings. Mathematical modelling can provide helpful insight by describing the types of interventions likely to maximize impact on the population level and highlighting those gaps in our current knowledge that are most important for making such assessments. This review discusses the major contributions of TB transmission models in general, namely, the ability to improve our understanding of the epidemiology of TB. We focus particularly on those elements that are important to appropriately understand the role of TB diagnosis and treatment (i.e., what elements of better diagnosis or treatment are likely to have greatest population-level impact) and yet remain poorly understood at present. It is essential for modellers, decision-makers, and epidemiologists alike to recognize these outstanding gaps in knowledge and understand their potential influence on model projections that may guide critical policy choices (e.g., investment and scale-up decisions).

## 1. Introduction

Recent decades have seen renewed interest in tuberculosis (TB) research, notably in areas of diagnostic test development and novel treatment regimens for TB and multidrug resistant TB (MDR-TB) [1–3]. New advances bring great potential to reduce TB burden and mortality, but resources remain highly constrained in most TB endemic settings. Mathematical modelling can serve to estimate the impact of various interventions on outcomes of interest; they can provide helpful insight by describing the types of interventions likely to maximize impact on the population level and highlighting those gaps in our current knowledge that are most important for making such assessments [4–8]. While the term “mathematical modelling” is used to describe a variety of techniques, this review will focus on transmission models designed to assess or understand the population-level (epidemiological) impact of TB control interventions.

**Figure 1:**
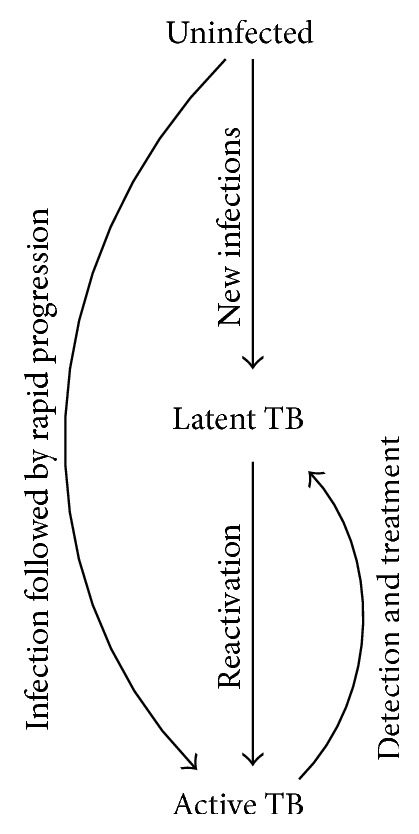
A simple epidemiological model of TB. Uninfected individuals that are exposed to TB can become infected with TB, which can result in either a long-standing infection that is asymptomatic and noninfectious (latent TB) or progress at some point (“reactivation”) to a condition that is infectious and generally symptomatic (active TB). Detection and effective treatment can cure active TB. For simplicity, some other important features of natural history of TB are not shown here (but are generally included in compartmental models of TB), including reinfection, spontaneous resolution (“self-cure”), and mortality.

The compartmental model, in which a population is divided into subpopulations or “compartments” on the basis of such characteristics as TB status, has historically been the most common form of TB mathematical model. Although other types of models, such as agent-based and network models, have been used to model specific transmission dynamics of TB [9–11], they are in general less frequently used in TB transmission models, where we are modeling airborne transmission of a chronic infection, compared to other infectious disease systems. In this outlook, we focus on compartmental models, which have been influential in modeling transmission dynamics of numerous infectious diseases, including droplet-borne respiratory diseases (e.g., influenza), sexually transmitted infections, and vector-borne diseases [12, 13]. The prototypical “SIR” model divides the population into susceptible (S), infected (I), and recovered (R) compartments, and transmission dynamics are described using rates of flow between these compartments. Given the complexities of TB pathology and the presence of a potentially long latency, compartmental models of TB are typically modified reflecting TB pathology, relevant context, and the research question of interest.  Figure 1 depicts a simplified compartmental model for TB transmission, in which the population is subdivided into compartments of individuals who have never been infected with TB, those who have been infected but are not currently infectious (latent TB), and those who are actively infectious and symptomatic. By evaluating the rates at which people flow from one compartment to another under different scenarios, such models can provide insight about not only the direct effects of those interventions on those who receive them, but also the indirect effects that occur through a reduction in transmission to the population as a whole.

Here, we use the compartmental model as a tool to highlight a major contribution of TB transmission models in general, namely, the ability to improve our understanding of the epidemiology of TB. We focus particularly on those elements that are important to appropriately understand the role of TB diagnosis and treatment (i.e., what elements of better diagnosis or treatment are likely to have greatest population-level impact) and yet remain poorly understood at present. It is essential for modellers, decision-makers, and epidemiologists alike to recognize these outstanding gaps in knowledge and understand their potential influence on model projections that may guide critical policy choices (e.g., investment and scale-up decisions). Only through such shared understanding can epidemiologists direct data collection efforts at the highest-yield targets, decision-makers understand both the value and limitations of model-based projections, and modelers seek to refine their tools in response to emerging data and policy needs.

## 2. Modelling Transmission

### 2.1. Infectiousness over Time

A key advantage of transmission models is that they incorporate the process of infection; in other words, interventions that lead to faster diagnosis of TB also benefit the population by reducing transmission [14]. However, the process of transmission is also an area of great uncertainty in TB. A commonly used measure to describe infectiousness is the effective reproductive ratio (*R*_*e*_), which represents the average number of secondary cases arising from a primary case of active TB in a population with its existing level of immunity. Notably, *R*_*e*_ depend on both the process of generating infectious particles and social mixing or contact patterns within the host population.

The impact of a given diagnostic intervention on TB transmission will depend on its effect on *R*_*e*_, as it is deployed in the population. Our ability to estimate the number of effective secondary cases produced by each index case and how a diagnostic intervention may reduce this number is therefore critical. However, accurately assessing the reproductive ratio for TB, much less the effect of a diagnostic intervention on that ratio, requires a better understanding of the context in which TB transmission occurs. Unfortunately, we currently lack a tool that can reliably detect recent TB transmission or infection: tests for latent TB infection do not differentiate between recent and remote infection, and tests for active TB likewise do not differentiate the timing of initial transmission leading to infectiousness [15, 16]. Unlike acute infections (e.g., influenza, measles, and diarrheal illness), the time from infection to disease in TB can be as short as a few months, or as long as decades. Even animal models are limited in this regard; with the exception of nonhuman primates, animal models including mice, guinea pigs, and rabbits do not approximate the human latent TB infection phase [17], and there is no animal model for human social interactions.

The effective reproductive ratio for TB in different populations depends on three processes: the generation of infectious particles, contact between infectious individuals and other members of society, and susceptibility of the host population [7]. The clinical course of TB infection and disease comprises a spectrum that is dynamic over time and varies between and within individuals [18] and includes elements of infectiousness, symptom burden, and changing social interactions (e.g., staying home when sick) that are all poorly understood. Better data to inform these dynamic processes would help to improve our estimates of the impact expected from different TB diagnostic interventions, and mathematical models can highlight which data elements are most critical [19].

### 2.2. Generation of Infectious Particles

As TB is an airborne disease, generation of infectious particles (“droplet nuclei”) is essential to transmission [20]. This process depends on both the infectious agent (e.g., ability to survive in small particles and to infect lung alveolar epithelium when inhaled) and the host (e.g., generation of particles sufficiently small to remain airborne and that contain infectious organisms). The presence of visible* M. tuberculosis* bacilli on microscopic examination of sputum (sputum smear status) is a correlate of infectiousness, but individuals with smear-negative TB may still contribute a substantial proportion of TB transmission on the population level [21]. This has important implications for diagnosis, which traditionally rests on sputum smear microscopy as an integral part of the diagnostic algorithm. For novel diagnostic tests to have important impact on TB epidemiology, they must improve upon sputum smear, in terms of its ability to identify those cases that contribute to community transmission. Whole genome sequencing is now frequently used in high-income countries to identify transmission clusters and super-spreaders [22–25], but these evaluations occur post hoc and only in lower-burden or research settings. Understanding how other diagnostic interventions are likely to alter the total number of infectious particles generated by a single representative individual with active TB may help to understand the likely impact of those interventions on the population level.

**Figure 2:**
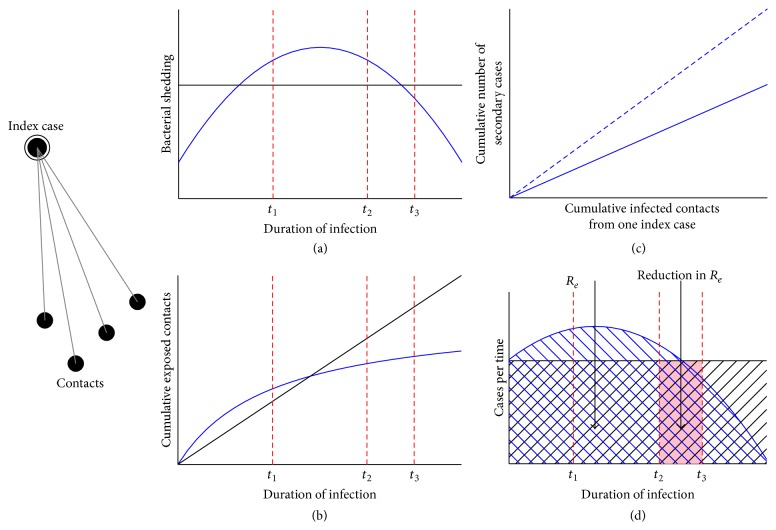
(a) The rate of bacterial shedding over the duration of infection may be a constant function (black line) or change over time (blue line). (b) The cumulative number of contacts exposed to TB over the duration of infection may increase linearly over time (black line) or may plateau as contact pool becomes saturated or patient is too ill to circulate in the community. The potential impact of a novel intervention may depend on this assumption; given a linear increase, earlier intervention (*t*_1_) would be likely preferred. While given the second curve with only a small increase and plateau, the impact between intervening at *t*_1_ and *t*_2_ might not be as great; therefore other factors including cost-effectiveness may come into play. (c) The cumulative number of secondary cases resulting from one index case in relation to the number of cumulative infected contacts: there are factors associated with bacterial virulence and host susceptibility that impact the rate of progression from infection to disease. This rate may be steeper among immunosuppressed contacts, for example, (dotted line) compared with immunocompetent contact (solid line). (d) The effective reproductive number (*R*_*e*_) is the number of secondary cases generated over a given time period. Bacterial shedding, contact mixing pattern, and bacterial and host susceptibility all contribute to the overall rate of secondary cases generated over time; depending on what assumptions are made these rates could be thought to stay constant over time or vary, perhaps tapering off over the duration of infection. By reducing the time to diagnosis and treatment initiation we hope to reduce the number of secondary cases but the amount of impact depends on assumptions around the shape of the curve over time. The hashed area represents the secondary cases generated from one index case while the shaded area represents the potential reduction in secondary cases given an intervention at *t*_2_. Figure adapted with permission from Dowdy et al. [7].

Within the context of diagnostic interventions, it becomes important to understand how both the generation of infectious TB particles and the rate of contact with susceptible individuals change over time with the evolving TB disease course (Figures 2(a) and 2(b)). Assumptions relating to these processes have important implications for the potential impact of diagnostic interventions [7]. For example, if diagnostics are deployed in such a way that most infectious contacts have already occurred by the time new diagnostic tests can be accessed, the impact of those novel tests on TB incidence will be limited—even if the tests are perfectly sensitive and specific. Similarly, if diagnostic tests are implemented without the infrastructure necessary to link people who test positive to appropriate treatment, the infectious course will remain unaltered. Specifically, Dowdy et al. used a model that included subclinical TB disease phase and found that ignoring the possibility of infectiousness prior to seeking care resulted in models that could overestimate the impact of passive diagnostic testing (i.e., testing that relies on symptomatic presentation by patients) by 50% or more [7]. This model demonstrated that active case detection of prevalent cases in the community is likely to have greater impact on the duration of infectiousness, and thus on TB incidence, than passive diagnosis. It also showed that estimates of the relative impact of different diagnostic strategies depend critically on improving our understanding of when in the disease process TB transmission occurs.

### 2.3. Contact between Infectious Individuals and Other Members of Society

Generation of an infectious particle will only cause transmission of TB if that particle contacts the lung epithelium of another individual. Such contact depends on social mixing patterns between people with active TB and other members of society. For example, if an index case only has extended exposure to household contacts, close friends and family there may be a point in time where the cumulative number of infected contacts reaches a plateau, with no or very few new contacts exposed (Figure 2(b)). In such a scenario, increasing levels of bacterial shedding from the index case may not result in more infections if the pool of susceptible contacts has reached saturation. Similarly, low levels of bacterial shedding during early onset of the disease, even prior to the patient recognizing any symptoms, may account for a significant proportion of transmission events if the duration of that period is long and characterized by more frequent airborne contacts (e.g., if people continue to work and interact with society during that time). Like the ability to generate infectious particles, the trajectory of social contact is likely to vary between individuals and across settings, and the point in that trajectory at which diagnostic interventions are deployed will determine the population-level impact of those interventions. Many models assume the simplest case (constant rate of contact over time), but the number of susceptible contacts most likely declines over time unless index cases are hospitalized, imprisoned, or otherwise introduced into a new population. This phenomenon likely decreases the potential impact of passive diagnostic testing, as most transmission events may occur early in the disease course. Kasaie et al. [10] employed agent based models to explore scenarios where transmission was dominated by either community or household transmission and the potential impact of household based contact tracing. The authors found that 75–95% of household infections would have occurred prior to the diagnosis of the focal case in the household, and, hence, household contact tracing by itself was unlikely to be transformative in terms of TB epidemiology. Better understanding the degree to which contact rate changes over time may be essential for better estimating the impact of novel diagnostic tests for TB.

### 2.4. Susceptibility of the Host Population

A third important determinant of the rate of TB transmission over time, and thus of the impact of diagnostic testing, is the susceptibility of the host population to developing active TB after a potentially infectious contact. This concept of susceptibility therefore encompasses both susceptibility to infection and susceptibility to progression if infection occurs. While not necessarily intrinsic to host susceptibility, bacterial strain virulence also plays an important role [26, 27]. Further determinants of TB susceptibility, including HIV, older age, diabetes, smoking, and malnutrition, have been described in the literature; however, the degree to which these determinants of susceptibility overlap with potential transmission events is only now becoming understood [28]. In settings where many infectious contacts occur with individuals of higher susceptibility profiles, *R*_*e*_ will be substantially higher than if those contacts occur with less susceptible people, as depicted in Figure 2(c). Indeed, one of the major reasons for the dramatic declines in TB incidence seen throughout much of the Western world is likely a reduction in the susceptibility profile of the population, while the TB rise in Africa is driven by the HIV epidemic [29].

### 2.5. Sensitivity and Uncertainty Analyses

Transmission modelling results are often accompanied by sensitivity and uncertainty analyses, which can achieve two important goals. First, some parameters used in the model can contain large uncertainty or variability in the estimates, arising from either the dearth of high quality data or the variability of estimates derived from different sources. Uncertainty analyses help give readers perspective on how uncertainty in model inputs might translate into uncertainty in model results. Second, sensitivity analyses of the model input parameters can provide critical information on which model parameters have the most influence on model results. Typical sensitivity analyses include one-way and multivariate sensitivity analyses. In one-way sensitivity analysis, one observes changes in the model outcome as a result of change in a single focal parameter, holding other parameters constant. In contrast, in a multivariate sensitivity analysis, one varies all or most of the model parameters over selected ranges and computes the model outcomes. By analyzing the correlation between the model outcome and a given parameter, one can assess the role of the parameter in the outcome [30]. Sensitivity and uncertainty analyses also do not address uncertainties arising from uncertainty or variability in the model structure.

## 3. The Role of Modeling: TB Diagnostics

### 3.1. Background

The field of TB diagnostics has seen major growth in the last decade, and many novel technologies are now available for use, with even more in the pipeline. Arguably the greatest technological breakthrough in TB control over the past decade has been a new diagnostic test: Xpert MTB/RIF, a molecular test for TB and rifampin resistance capable of providing results in two hours with minimal human resource requirement [31]. Important questions are now arising as policymakers must consider implementing rapidly expanding options for TB diagnostics, while drawing on limited budgets.

Mathematical modeling can help policymakers understand the potential population-level impact and cost-effectiveness associated with implementing novel diagnostic tests. Importantly, models can consider a wide variety of settings, populations, and diagnostic algorithms to help inform the “right diagnostic approach for the right setting”—in other words, helping us to understand what population characteristics will lead to different approaches having greater or less impact. Whereas decision-makers often express an interest in models that will project the future under different implementation scenarios, understanding the factors that drive the impact of different diagnostic approaches is often a more important long-term goal—and models are uniquely positioned to provide this kind of insight.

Lin et al. have conducted modelling studies to estimate the potential impact of new diagnostic tools using detailed models of the diagnostic pathway and integrating operational and dynamic transmission models [32, 33]. This work compared the reduction in incidence, prevalence, and mortality of pulmonary TB achieved by new diagnostic tools against a baseline case of sputum smear microscopy. They demonstrated the importance of including operational context and the diagnostic pathway in models evaluating novel diagnostics; for example, the epidemiologic impact of a new more accurate tool was greatest in settings where access to tuberculosis care was good but existing diagnostic strategies have poor sensitivity and was less dependent on its relative performance. These models can be informative both for guiding decisions around novel tools and the impact of alternative diagnostic pathways; however useful projections rely on capturing the relevant structure of the transmission dynamics and diagnostic pathway at work.

Matching the appropriate diagnostic test and algorithm to a given setting is an important task but one that requires understanding of interactions between population epidemiology, test characteristics, operational considerations (e.g., feasibility of scale-up), and resource requirements. Depending on the interventions being considered, different assumptions may be required—such as those relating to test accuracy (e.g., sensitivity and specificity), use (e.g., diagnostic algorithm and purpose of the test), underlying population, and costs. Improved understanding of which assumptions are most critical to specific decisions regarding TB diagnostics can help the TB control community direct data-gathering efforts and make more informed decisions in the future.

### 3.2. Summary: Infectiousness over Time and Implications for Models of Diagnostic Interventions

Figure 2(d) depicts the rate of transmissions that will ultimately lead to a secondary case of infectious TB, over the disease course of an index case. Upon contact with a diagnostic intervention (*t*_2_) that results in treatment that would otherwise be delayed (*t*_3_), the shaded area between those two times and under the curve represents the reduction in *R*_*e*_ achieved. This figure demonstrates that the potential impact of diagnostic interventions depends on not only their accuracy but also how early in the disease course they can be deployed and the shape of the “transmission curve” before versus after contact with the diagnostic intervention. Understanding this shape is key to the development of accurate models of TB diagnostics and incorporates the elements discussed above: generation of infectious particles, contact, and susceptibility. The duration between *t*_2_ and *t*_3_ likewise depends on characteristics of the health system and patients' interactions with that system [32, 33]. The potential impact of novel diagnostics can be attenuated not only by flaws in the test, but also by delays in care seeking, diagnosis, and treatment (i.e., longer time to *t*_2_ in Figure 2(d)).

In summary, although active case finding is a stated goal, at present we must still rely on symptomatic presentation by patients for most TB diagnoses to occur. Mathematical models can help decision-makers understand the potential impact of novel TB diagnostic tests, but they also highlight the key data gaps that prevent us from being able to make more accurate, evidence-based projections. Those data gaps include insufficient knowledge about the trajectories of infectiousness, mixing, and population susceptibility over time, including how those trajectories are influenced by the pathogen, host, and health system. If we are to understand the population-level impact of novel TB diagnostics, the next wave of epidemiological data gathering must address these deficiencies in our current understanding.

## 4. The Role of Modeling: TB Drugs and Drug Resistance

### 4.1. Background

For the first time in many decades [34] new first-line drug regimens are being considered for treatment of TB. Some of these regimens make repurposed use of existing drug compounds (e.g., fluoroquinolones) and others use novel compounds (e.g., PA-824) [35–37]. The possibility of new first-line drug regimens for TB offers an opportunity to further investigate the dynamics of TB drug resistance, which will be a key consideration in the roll-out of any such regimen. Existing second-line regimens require prolonged, costly, uncomfortable treatment (often 24 months with up to 8 months of daily injections) with a much greater risk of side effects including neuropsychiatric effects, loss of hearing, and kidney failure [38–40]. Since such regimens are very challenging to complete, it is essential to limit our dependence on such regimen from a population perspective. However, transmission of resistance can spur a vicious cycle: as resistant strains of TB become more prevalent, they spread more rapidly, in turn increasing the transmission burden of drug resistant TB. Mathematical models can help guide decision-making and elucidate key knowledge gaps in this complex arena [41–46]. In the context of resistance to new first-line drug regimens, the role of modeling is particularly important for two different reasons.

First, drug resistance is a multifactorial process, and the emergence of drug resistance in a population setting depends on several underlying factors [47]. As with diagnostic considerations above, these include factors intrinsic to the pathogen, including genetic barriers to drug resistance [48–52]; factors related to contact/transmission, including TB prevalence (i.e., transmission burden) and treatment success [48]; and factors related to host susceptibility, including HIV prevalence [53]. While we have developed a rudimentary understanding of some of these factors, models can help to demonstrate where more comprehensive data are necessary to understand the likely dynamics of TB drug resistance under pressure from new drugs and new regimens.

Second, novel regimens are, by definition, implemented in settings that have no data as to how the regimen will affect TB dynamics on the population level. In such data-free situations, models are essential for making “first-pass” projections and for informing which data are the most essential to collect. Mathematical models can shape our understanding by extrapolating relevant information from existing epidemics (e.g., of MDR-TB). Such understanding can form a basis for well-informed, targeted policies for appropriate deployment of new regimens, augmentation of those regimens with drug susceptibility testing (DST), and ongoing collection of epidemiological data.

### 4.2. A Simple Model of Drug Resistance in TB

**Figure 3:**
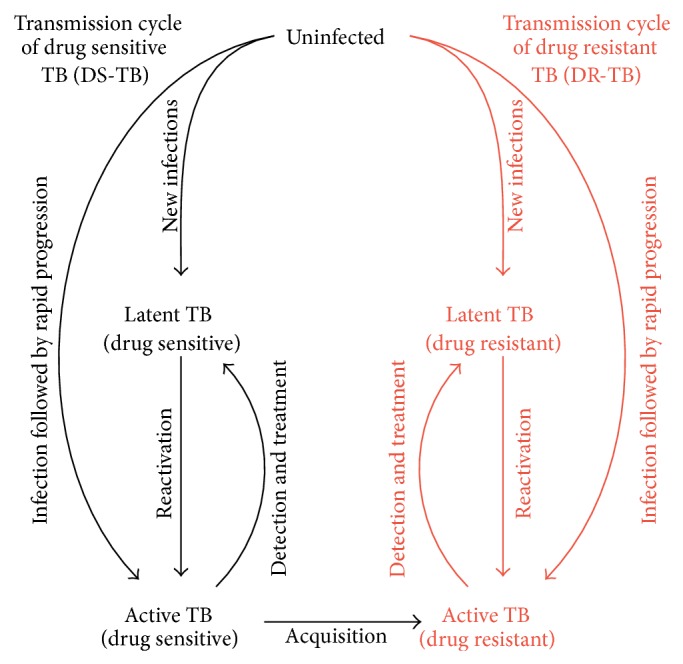
A simple epidemiological model of drug resistant (DR-)TB. This model divides the transmission cycle of TB into two arms: transmission of DS-TB and DR-TB (which is shown in red). For simplicity and comparability, the transmission cycle of DR-TB is structurally similar to DS-TB. The difference between DS-TB and DR-TB can be characterized by difference in rates of transition between different compartments. (E.g., if the transmission fitness of DR-TB is less than that of DS-TB, the rates of new infections of DR-TB are lower compared to DS-TB.) The acquisition of drug resistance during treatment resulting from de novo mutations is a primary way in which drug resistance enters the population. Subsequently, drug resistance can spread via transmission events. Increasing the rate at which DR-TB is successfully diagnosed and treated (e.g., through drug susceptibility testing and regimen modification) can be modeled as an increase in the flow from compartment “Active DR-TB” back to “Latent DR-TB” (or, in an alternative formulation, back to uninfected).

In the absence of detailed data, a reasonable approach to understand the emergence and transmission dynamics of drug resistance is to construct simplified transmission models of drug resistant TB. One such simplified model might subdivide TB strains into two categories: those that are sensitive to a hypothetical first-line TB drug regimen (DS-TB) and those that are resistant (DR-TB) [46, 54, 55]. This is illustrated schematically in Figure 3. In this framework, resistance is acquired during treatment via de novo mutations and propagated via ongoing transmission thereafter. In comparison to the model shown in Figure 1, the model in Figure 3 now consists of two arms, representing transmission cycles of DS-TB and DR-TB. This framework allows for exploration of different aspects of DR-TB including the relative fitness of DS-TB and DR-TB, relative treatment success, and acquisition of resistance during treatment. These characterizations can be informed by data from existing experience with other resistant strains (e.g., MDR-TB) and expanded to consider additional illustrative scenarios.

### 4.3. Reproductive Fitness of Drug Resistant TB

Analogous to the effective reproductive ratio *R*_*e*_ discussed above, we can develop a similar concept of *R*_*e*,dr_ as applied to drug resistant strains. This quantity is the average number of secondary DR-TB infections resulting from a single primary DR-TB infection, in the presence of an existing DS-TB epidemic. This effective reproductive ratio can serve as a theoretical basis of the reproductive fitness of the resistant strains—resistant strains with *R*_*e*,dr_ of 1 have comparable potential as DS-TB to spread throughout the population, whereas those with *R*_*e*,dr_ < 1 have less potential to spread, and those with *R*_*e*,dr_ > 1 have the potential, given sufficient time, to replace DS-TB in the population.

**Figure 4:**
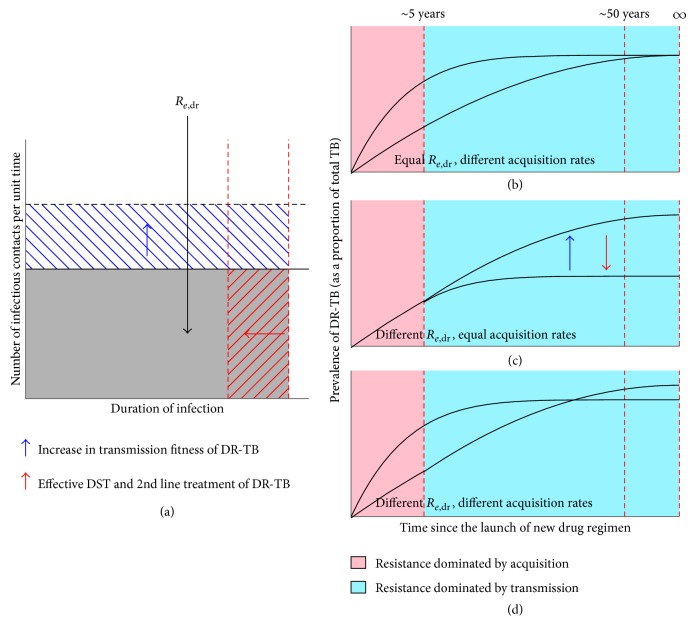
Proliferation of drug resistance following the launch of new first-line drug regimen. (a) The effective reproductive ratio of DR-TB (*R*_*e*,dr_) is the expected number of secondary cases of active, resistant TB resulting from a single case of DR-TB (shown as the grey shaded area). An increase in the relative transmission fitness of DR-TB (e.g., due to compensatory mutations; shown by the blue arrow) increases *R*_*e*,dr_ (shown by the blue hatched area). Shortening the average duration of DR-TB infections (e.g., by deployment of DST, and effective second-line treatment; shown by the red arrow) decreases *R*_*e*,dr_ (shown by the red hatched area). However, the rate of acquisition of drug resistance (e.g., due to de novo mutations against drugs in the treatment regimen) does not factor in the calculation of *R*_*e*,dr_ (b, c, and d). The trajectories of the prevalence of DR-TB just following the launch of a hypothetical new drug regimen are affected by both the acquisition rates and the *R*_*e*,dr_ of DR-TB, but their effects will be more pronounced at different time periods. Acquisition-driven drug resistance is expected to be more frequent in the first 5 years (pink area), while transmission-driven TB relatively later (blue area). (b) For two hypothetical DR-TB strains with similar *R*_*e*,dr_, but different acquisition rates, we may observe difference in their prevalence in the short term, but over time they are expected to result in similar levels of resistance. (c) In contrast, for strains with similar acquisition rates, but different *R*_*e*,dr_, we may not observe significant difference in their prevalence in the short term, but the levels of drug resistance can diverge significantly. Factors that affect *R*_*e*,dr_ will affect the trajectories of DR-TB prevalence—for example, deployment of DST that achieve reduction in average duration of infection (red arrow) can reduce prevalence of DR-TB over longer term. (d) DR-TB strain with larger acquisition rate and smaller *R*_*e*,dr_ is expected to be more prevalent over the short term compared to a strain with lower acquisition rate and higher *R*_*e*,dr_, but the prevalence of DR-TB is flipped between two hypothetical strains over longer term. Hence, short term prevalence of DR-TB alone may not be a reliable predictor of the prevalence over longer term. Figures are only illustrative and not drawn to scale.

The relationship between key drivers of drug resistance and *R*_*e*,dr_ can inform the relative importance of each factor in influencing the long-term emergence of DR-TB. One revealing insight afforded by this exercise is that the rate of acquisition of drug resistance does not affect the effective reproductive ratio. This implies that transmission of DR-TB is much more important than the acquisition of resistance during treatment in terms of affecting long-term trajectories of DR-TB after scale-up of a new first-line regimen. Just as the product of transmission rate (cases per time) and the effective duration of infection make up the effective reproductive ratio *R*_*e*_ (see Figure 2), the relative transmission rate and relative duration of infectiousness of DR-TB versus DS-TB determine *R*_*e*,dr_ relative to *R*_*e*,ds_, as shown in Figure 4(a).

### 4.4. The Trajectories of DR-TB after Launch of a New First-Line Drug Regimen

Trajectories of the prevalence of DR-TB produced by simulating this model can further elucidate the role of these different drivers of TB drug resistance over time. During the first years following regimen introduction (first 5 years after the launch of new first-line drug regimen), acquisition during treatment is a more important determinant of DR-TB at the population level than is transmission, whereas transmission becomes more important in later years. This shift reflects the changing balance between a constant risk of acquisition per treatment episode and a transmission risk that is proportional to an expanding pool of prevalent DR-TB.

### 4.5. Consequences of Public Health Interventions

The method by which new drug regimens are rolled out, and in particular the role of concomitant drug susceptibility testing, will ultimately shape the trajectory of DR-TB prevalence over time. In particular, the effect of various implementation strategies on the effective duration of DR-TB (i.e., shortening that duration by speeding the process of diagnosis and initiation of appropriate treatment) will be critical. Individuals with active DR-TB that remain undetected or untreated will fuel drug resistance through ongoing transmission. Early detection of drug resistance via drug susceptibility testing (DST) and rapid initiation of effective therapy will be key to controlling this spread. Surveillance data on the prevalence of DR-TB during the first few years after the launch of new first line regimens will reflect acquisition rather than transmission burden and therefore may not be indicative of longer term trajectories. It is therefore important to collect not only surveillance data, but also data to inform the relative transmission fitness and treatment success of DR-TB. As with models of new diagnostic tests, models of new drug regimens can therefore inform not only appropriate decision-making with respect to scale-up of interventions, but also the epidemiological data-gathering efforts that are most likely to enhance our ability to project impact at the population level.

### 4.6. Summary: Models of Drug Resistance under New First-Line Regimens

Emergence of drug resistance is a multifactorial process that includes the interplay between pathogens (e.g., genetic barriers to resistance), contact patterns, and duration of infectiousness. Two of the most important drivers of drug resistance are the relative competitive fitness of DR-strains and the relative treatment success (which in turn determines the relative duration of disease). Regarding competitive fitness, lab experiments (e.g., competition assay) may provide basic insight, but fitness in the lab may not correlate with fitness as transmitted via aerosols between host systems with heterogeneous mixing [56, 57], different pathogen characteristics [58–60], and in the possible presence of compensatory mutations [61, 62]. Regarding treatment success, program data can provide a helpful start, but detailed data on relapse after treatment and duration of infectiousness for those failing treatment are also critical. Mathematical models again play an important role in understanding the system of DR-TB, deploying appropriate interventions (e.g., DST), and driving the collection of key epidemiological data.

## 5. Conclusions

Mathematical models continue to provide valuable insight into potential impact and cost-effectiveness of strategies to improve both diagnosis and treatment of TB. While often expected to provide projections of alternative futures, their greater contribution may lie in informing decisions as to the best path given existing data, providing better understanding of the key drivers of impact, and informing more relevant data collection efforts in the future. In both of the systems described here (diagnostics and drug regimens), host, pathogen, and health system factors combine to drive infectiousness, mixing patterns, and population susceptibility. Models can demonstrate how the interplay between these elements drives TB epidemiology under the influence of novel interventions; one important way of achieving this aim is by describing interventions' effects on the effective reproductive ratio *R*_*e*_. Future efforts to control TB will benefit from increased collaboration between epidemiologists, decision-makers, and modellers. Models of TB diagnostics and novel drug regimens represent two realms in which such discussions are starting to take place.
